# Characterization of the Hypothalamic-Pituitary-Adrenal-Axis in Familial Longevity under Resting Conditions

**DOI:** 10.1371/journal.pone.0133119

**Published:** 2015-07-20

**Authors:** Steffy W. Jansen, Ferdinand Roelfsema, Abimbola A. Akintola, Nicole Y. Oei, Christa M. Cobbaert, Bart E. Ballieux, Jeroen van der Grond, Rudi G. Westendorp, Hanno Pijl, Diana van Heemst

**Affiliations:** 1 Department of Gerontology and Geriatrics, Leiden University Medical Center, Leiden, The Netherlands; 2 Department of Endocrinology and Metabolism, Leiden University Medical Center, Leiden, The Netherlands; 3 Developmental Psychology (ADAPT-lab), University of Amsterdam, Amsterdam, The Netherlands; 4 Amsterdam Brain and Cognition (ABC), University of Amsterdam, Amsterdam, The Netherlands; 5 Department of Radiology, Leiden University Medical Center, Leiden, The Netherlands; 6 Department of Clinical Chemistry and Laboratory Medicine, Leiden University Medical Center, Leiden, The Netherlands; 7 Leiden Institute for Brain and Cognition (LIBC), Leiden University, Leiden, The Netherlands; 8 Department of Public Health, University of Copenhagen, Copenhagen, Denmark; University of Alabama at Birmingham, UNITED STATES

## Abstract

**Objective:**

The hypothalamic-pituitary-adrenal (HPA)-axis is the most important neuro-endocrine stress response system of our body which is of critical importance for survival. Disturbances in HPA-axis activity have been associated with adverse metabolic and cognitive changes. Humans enriched for longevity have less metabolic and cognitive disturbances and therefore diminished activity of the HPA axis may be a potential candidate mechanism underlying healthy familial longevity. Here, we compared 24-h plasma ACTH and serum cortisol concentration profiles and different aspects of the regulation of the HPA-axis in offspring from long-lived siblings, who are enriched for familial longevity and age-matched controls.

**Design:**

Case-control study within the Leiden Longevity study cohort consisting of 20 middle-aged offspring of nonagenarian siblings (offspring) together with 18 partners (controls).

**Methods:**

During 24 h, venous blood was sampled every 10 minutes for determination of circulatory ACTH and cortisol concentrations. Deconvolution analysis, cross approximate entropy analysis and ACTH-cortisol-dose response modeling were used to assess, respectively, ACTH and cortisol secretion parameters, feedforward and feedback synchrony and adrenal gland ACTH responsivity.

**Results:**

Mean (95% Confidence Interval) basal ACTH secretion was higher in male offspring compared to male controls (645 (324-1286) ngl/L/24 h *versus* 240 (120-477) ng/L/24 h, P = 0.05). Other ACTH and cortisol secretion parameters did not differ between offspring and controls. In addition, no significant differences in feedforward and feedback synchrony and adrenal gland ACTH responsivity were observed between groups.

**Conclusions:**

These results suggest that familial longevity is not associated with major differences in HPA-axis activity under resting conditions, although modest, sex-specific differences may exist between groups that might be clinically relevant.

## Introduction

The hypothalamic-pituitary-adrenal (HPA)-axis is the most important neuro-endocrine stress response system of our body which is of critical importance for survival. Different stressors can trigger the neurons in paraventricular nuclei of the hypothalamus to secrete corticotrophin-releasing-hormone (CRH). CRH stimulates the pituitary gland to secrete adrenocorticotropic hormone (ACTH), which binds to ACTH receptors on the adrenal gland and stimulates the secretion of glucocorticoids, of which cortisol is the most important[1]. Cortisol inhibits the HPA-axis via classical negative feedback mechanisms involving the hippocampus, hypothalamus and pituitary[1]. Tightly controlled regulation of hypothalamic-pituitary-adrenal (HPA)-axis responses is of importance for maintaining both mental and physical health, since both hyper and hypo-activity of the HPA-axis are linked to disease states[1, 2]. If untreated, patients with severe Cushing's syndrome, who is characterized by cortisol excess, and patients with adrenal insufficiency, which are cortisol deficient, have a remaining life expectancy of a few years, while restoration of the HPA-axis recues health and substantially increases remaining life expectancy. In addition, diabetes, hippocampal damage[3] and hypertension[4] are associated with a blunted or absent cortisol response after waking up, and higher cortisol levels in the evening are associated with increased blood pressure and insulin resistance[5–7].

The Leiden Longevity Study (LLS) was designed to identify genetic mechanisms underlying healthy familial longevity[8] and comprises nonagenarians with at least one nonagenarian sibling, their offspring and the offspring’s partners serving as an age-matched control group. To assess whether our recruitment strategy had resulted in enrichment for familial longevity, we compared the mortality rates of included first degree relatives with those of their respective birth cohorts. We found that all groups of first degree relatives (including parents, additional siblings and offspring) had on average a 30% lower mortality rate compared to their birth cohorts, illustrating a successful enrichment for familial longevity[8]. In line, compared to their partners (controls), already at middle age, offspring from nonagenarian siblings (offspring) had lower prevalence of cardiovascular and metabolic diseases[9] and better cognitive performance also after adjustment for potential confounders, including myocardial infarction and type 2 diabetes[10]. Moreover, among non-diabetic participants, offspring compared to their partners, had better glucose tolerance[11] and higher insulin sensitivity[12]. It is unknown which mechanisms underlie the favorable cardiovascular and metabolic health profile and the survival advantage displayed by the offspring. Since changes in HPA-axis activity have been associated with the adverse metabolic and cognitive changes that typify partners as compared to offspring, diminished basal activity of the HPA axis may a potential candidate mechanism underlying healthy familial longevity. In previous studies in the LLS, we found in a single morning blood sample no significant difference in cortisol concentrations between offspring and controls[13]. However, when taking multiple saliva samples in the morning and evening, the area under the curve (AUC) of morning and evening salivary cortisol concentrations were slightly lower in the offspring[14].

Therefore the purpose of this study was to investigate whether offspring from long-lived siblings enriched for familial longevity, compared to controls, had differences in HPA-axis activity and/or regulation, reflected by different plasma ACTH and serum cortisol concentration profiles over 24 h or distinct hormonal interactions. In the present study we collected blood samples every 10 minutes, which allows for detailed deconvolution analysis of the 24-h ACTH and cortisol concentration profiles to estimate basal, pulsatile and total secretion of ACTH and cortisol over 24 h as well as specific secretion parameters. In addition, we studied the regularity of ACTH and cortisol secretion using approximate entropy (ApEn). Furthermore, we assessed ACTH-cortisol feedforward and cortisol-ACTH feedback synchrony using cross-ApEn. Finally, we assessed adrenal gland sensitivity to ACTH in offspring and controls by modelling an endogenous ACTH-cortisol dose-response relationship.

## Subjects and Methods

### Study population

Participants were derived from the Leiden Longevity Study (LLS), a family based study consisting of 421 families with at least two long-lived siblings (men ≥ 89 year, women ≥ 91 year) of Dutch descent, without any selection on demographics or health[8, 9]. For the current study (Switchbox), 20 offspring from long-lived siblings and 18 controls (partners from offspring) from the LLS were included who met the inclusion criteria of being middle-aged (55–77 years) and having a stable body mass index (BMI) between 19 and 33 kg/m^2^. Exclusion criteria were: any significant chronic, renal, hepatic or endocrine disease, mild depression (> 10 point for the Geriatric depression scale-30) or medication use known to influence any hormonal axis including estrogen replacement therapy for women, anaemia (haemoglobin < 7.1 mmol/L), fasting plasma glucose > 7 mmol/L, recent blood donation or trans-meridian flights, smoking addiction, use of more than 20 units of alcohol per week, or extreme diet therapies. To enhance the contrast in familial longevity between groups, controls with a nonagenarian parent who had one or more nonagenarian siblings were excluded (based on telephone questioning). The Switchbox protocol was approved by the Medical Ethical Committee of the Leiden University Medical Center and was performed according to the Helsinki declaration. All participants gave written informed consent for participation after full explanation of the purpose and nature of all procedures used.

### Clinical protocol

Participants were admitted to the research center at 0800 h, where a catheter was placed in a vein of the forearm of the non-dominant hand. After approximately an hour rest, blood sampling started at 0900 h. During 24 h, every 10 minutes 1.2 mL of blood was collected in a K3-EDTA tube and 2 mL in a serum separator (SST) tube. In total 461 mL of blood was withdrawn from each participant. All participants received standardized feeding at three fixed times during the day (between 0900–1000 h, 1200–1300 h, and 1800–1900 h), each consisting of 600 kcal Nutridrink (Nutricia Advanced Medical Nutrition, Zoetermeer, The Netherlands). Light exposure was standardized and lights were switched off between 2300–0800 h. No naps were allowed and participants ambulated only to the bathroom. All participants were sampled in the same room.

### Assays and assay performance

All measurements were performed at the department of clinical chemistry and laboratory medicine (AKCL) of Leiden University Medical Center, which is accredited according to CCKL (National Coordination Committee for Quality Assurance for Health Care Laboratories in The Netherlands). Cortisol was measured using an ECLIA assay on a Modular E170 analyser from Roche (Roche Diagnostics, Almere, The Netherlands), ACTH and DHEAS on an Immulite 2000 Xpi analyser (Siemens Healthcare diagnostics, The Hague, The Netherlands) and HbA1c on a Primus Ultra 2 HPLC analyser (Trinity Biotech, Bray, Ireland), using boronate affinity separation. For each participant, all samples from one time series were measured within the same lot number and in the same batch. For this study, the precision and quality of all assayed analytes met or surpassed the level of desirable quality specifications[15]. For cortisol Randox controls (Cat. Nr. I/1160EC and 3/1165EC) were used and overall coefficients of variation (CV) for cortisol ranged between 2.4–5.1%, which was well below the desirable CV of 10.5%. For ACTH two levels of controls were used (C2000LACCM1 and C2000LACCM2) and the CV ranged between 3.8–7.7%, which was well below the desirable CV of 10%. In our laboratory the reference range for is ACTH is 3–75 ng/L, for cortisol 0.1–0.6 μmol/L, for HbA1c 20–42 mmol/mol Hb.

### Deconvolution analyses

Each hormone concentration time series was analyzed using an automated deconvolution method. This method was validated using frequent blood sampling, and simulated pulsatile time series, as previously described[16–18]. Outcome parameters included number of pulses per 24 h, mean pulse mass, basal and pulsatile secretion, hormone half-lives, pulse mode (time to reach the maximal value) and the Weibull gamma value, representing the regularity of the statistically significant hormone pulses.

### Approximate entropy (ApEn)

ApEn is a scale- and model-independent univariate regularity statistic used to quantitate the orderliness (subpattern consistency) of serial stationary measurements. Mathematical models and feedback experiments have established that pattern orderliness monitors feedback and/or feed-forward interactions within an interlinked axis with high sensitivity and specificity, both greater than 90%[19]. Reduced pattern regularity typifies hormone secretion in puberty and aging, during diminished negative feedback or fixed exogenous stimulation, and by autonomous neuroendocrine tumors[20].

### Cross-ApEn

Cross-ApEn is a bivariate, scale-and model-independent two-variable regularity statistic used to quantitate the relative pattern synchrony of coupled time series[21]. Changes in the cross-ApEn of cortisol-ACTH reflect feedback synchrony and in the cross-ApEn of ACTH-cortisol reflect the feedforward synchrony with high sensitivity and specificity[22].

### ACTH-cortisol-dose response measurements

To explore adrenal gland sensitivity to ACTH in more detail, an endogenous ACTH-cortisol dose response curve was modelled. Details of the dynamic dose-response methodology were described in two previous papers[23, 24]. The goal was to relate time-varying plasma ACTH concentrations (input or effector) to time-varying cortisol secretion rates (output or response), based on fitted (deconvolved) ACTH concentrations and (deconvolved) cortisol secretion rates via the four-parameter (basal, potency, sensitivity and efficacy) logistic dose-response model modified to include a potency-down-regulated parameter and matching inflection time[25].

### Statistical analysis

S1 Dataset is the minimal anonymized dataset containing the individual participant data used for analyses. All analyses were done using linear regression analysis adjusted for age and BMI to investigate differences between offspring and partners. All data are presented as mean with standard error of the mean (SEM). Logarithmic transformation of data that were not normally distributed (basal, pulsatile and total secretion of ACTH and cortisol) was used to decrease the variation and these data are presented as a geometric mean with 95%-confidence interval (CI).

For all above-mentioned analyses, SPSS for Windows, version 20 (SPSS, Chicago, IL) was used. Graphs were made using GraphPad Prism version 5 (GraphPad, San Diego, CA) and Sigmaplot version 11 (Systat Software, Erkrath, Germany). *P* ≤ 0.05 was considered significant.

## Results

### Baseline characteristics

Baseline characteristics of offspring and controls are presented in Table 1 (for men and women combined and stratified for sex). Participants were selected on the basis of the age of their parents. Consequently, mothers of offspring were significantly older (men and women combined *P* < 0.001). Compared to controls, offspring were of similar age and BMI.

**Table 1:** Baseline characteristics of offspring from long-lived siblings and controls, in all participants and stratified for sex.

	All participants		Men		Women	
	Offspring n = 20	Controls n = 18	Offspring n = 10	Controls n = 10	Offspring n = 10	Controls n = 8
**Parental age**						
Mother (yr)	94.5 (89–97)	81.5 (77–88)	96.0 (88–98)	83.0 (77–88)	93.0 (89–97)	79.5 (68–88)
Father (yr)	89.5 (72–96)	78.0 (74–82)	89.5 (68–96)	77.0 (71–80)	89.5 (71–97)	80.0 (73–85)
**Demographics**						
Age (yr)	65.5 (5.4)	64.6 (4.9)	66.6 (6.4)	64.6 (4.0)	64.7 (4.4)	64.5 (6.1)
BMI (kg/m^2^)^a^	25.4 (4.0)	25.5 (3.9)	26.0 (3.4)	25.9 (3.2)	24.7 (4.6)	24.9 (4.8)
**Laboratory results**						
HbA1c (mmol/mol Hb)	34.6(1.5)	35.4 (2.0)	34.6 (1.9)	35.5 (1.8)	34.7(1.3)	35.3 (2.4)

All data are presented as the median with interquartile range or as the mean with standard deviation.

^a^BMI: Body Mass Index.

### Twenty-four hour hormone concentration profiles

**Fig 1:**
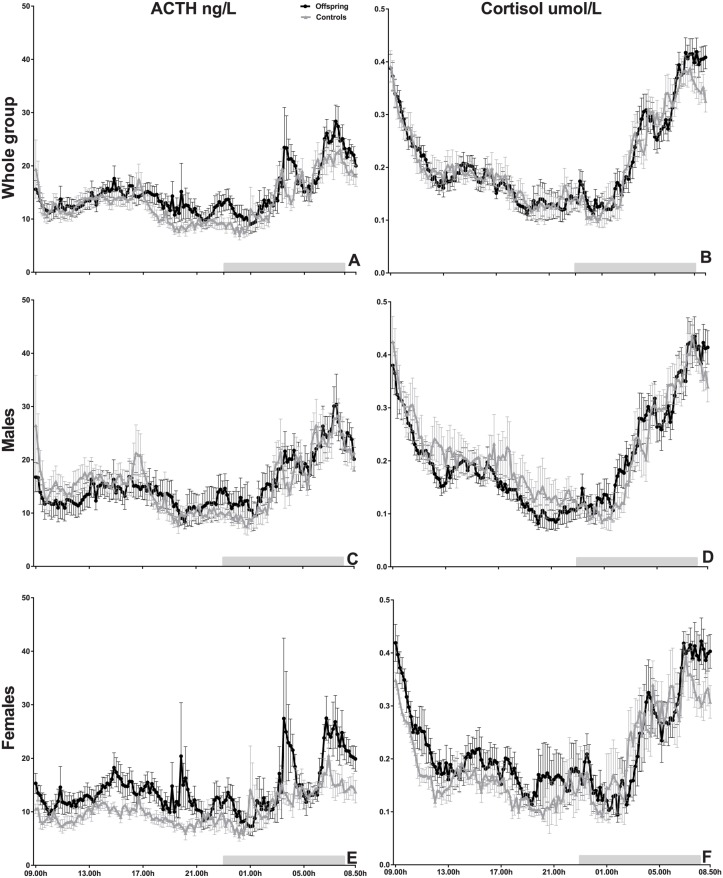
Mean 24-h concentration profiles of ACTH and cortisol in all participants and stratified for sex. The black dots represent hormone concentrations of 20 offspring and the grey triangles represent hormone concentrations of 18 controls every 10 minutes over a 24-h period for (A) ACTH and (B) cortisol. The black dots represent hormone concentrations of 10 male offspring and the grey triangles represent hormone concentrations of 8 male controls every 10 minutes over a 24-h period for (C) ACTH and (D) cortisol. The black dots represent hormone concentrations of 10 female offspring and the grey triangles represent hormone concentrations of 10 female controls every 10 minutes over a 24-h period for (E) ACTH and (F) cortisol. Error bars represents the standard error of the mean. Grey rectangle represents the night period (0000h-0700 h).

Mean 24-h plasma ACTH and serum cortisol concentration profiles are displayed in Fig 1. By visual inspection, ACTH concentrations seemed higher in offspring between 1700 and 0100 h, while there were no differences in cortisol concentrations (all participants, Fig 1A and 1B). In males no differences in 24-h ACTH concentrations were visible while cortisol levels during the day seemed lower in male offspring (Fig 1C and 1D). Female offspring seemed to have higher plasma concentrations of ACTH and serum concentrations of cortisol during the day (Fig 1E and 1F). However, mean plasma ACTH and mean serum cortisol concentrations did not differ between groups in any of these time periods (Table 2).

**Table 2:** Mean plasma ACTH and serum cortisol concentrations in all participants and stratified for sex.

	All participants			Men			Women		
	Offspring n = 20	Controls n = 18	P-value	Offspring n = 10	Controls n = 10	P-value	Offspring n = 10	Controls n = 8	P-value
**ACTH (ng/L)**									
24-h period	14.0 (11.8–16.5)	13.0 (10.9–15.6)	0.57	14.6 (11.4–18.8)	15.5 (12.0–19.9)	0.74	13.4 (10.8–16.6)	10.5 (8.3–13.4)	0.14
0900–1700 h	12.8 (10.7–15.3)	12.6 (10.5–15.2)	0.91	13.0 (10.0–17.0)	15.3 (11.7–20.0)	0.38	12.6 (10.1–15.8)	9.9 (7.7–12.8)	0.15
1700–0100 h	11.0 (9.3–13.2)	9.2 (7.7–11.1)	0.16	11.4 (8.7–14.9)	10.0 (7.6–13.0)	0.47	10.7 (8.3–13.9)	8.4 (6.3–11.2)	0.19
0100–0900 h	17.8 (14.8–21.3)	16.8 (13.8–20.3)	0.66	19.1 (14.7–25.0)	20.4 (15.6–26.7)	0.72	16.5 (13.0–20.9)	13.1(10.1–17.1)	0.19
**Cortisol (nmol/L)**									
24-h period	206 (188–226)	204 (186–225)	0.92	201 (177–227)	211 (186–239)	0.57	211 (181–245)	196 (166–233)	0.52
0900–1700 h	210 (187–236)	209 (184–236)	0.95	204 (171–244)	229 (191–274)	0.36	216 (185–253)	186 (156–222)	0.20
1700–0100 h	126 (106–152)	127 (105–154)	0.95	110 (86–142)	128 (100–164)	0.38	145 (110–192)	126 (92–173)	0.50
0100–0900 h	249 (251–302)	268 (243–296)	0.68	282 (254–313)	267 (240–296)	0.45	269 (227–320)	270 (223–326)	0.99

Data are presented as a geometric mean with 95% confidence interval.

Statistical significance was calculated with linear regression.

### ACTH and cortisol secretion

Results of the deconvolution analyses are displayed in Table 3. In men and women combined, there were no significant differences in basal, pulsatile and total ACTH and cortisol secretion between offspring and controls over 24 h. Mean (95% CI) basal ACTH secretion was higher in male offspring compared to male controls (645 (324–1286) ng/L/24 h *versus* 240 (120–477) ng/L/24 h, *P* = 0.05). When basal ACTH secretion was measured over 3 different time periods (0900–1700 h, 1700–2400 h and 2400–0800 h), it tended to be higher in male offspring, but did not reach statistical significance in one of the three time periods (S1 Table). Except for a lower basal cortisol secretion from 1700–2400 h in offspring, no differences were observed in cortisol secretion between groups over the three time periods in men (S1 Table). In women, there were no significant differences in basal, pulsatile or total ACTH or cortisol secretion between offspring and controls over 24 h (Table 3). When analyzed over the three time periods separately, no differences were observed between groups in women, except for a higher pulsatile ACTH secretion in offspring between 0900–1700 h (188 (141–251) ng/L *versus* 107 (78–148) ng/L, *P* = 0.02)) and a higher basal cortisol secretion from 1700–2400 h in the offspring (S1 Table).

**Table 3:** ACTH and cortisol secretion in all participants and stratified for sex.

	All participants			Men			Women		
	Offspring n = 20	Controls n = 18	P-value	Offspring n = 10	Control n = 10	P-value	Offspring n = 10	Controls n = 8	P-value
**ACTH**									
Basal (ng/L/24 h)	556 (360–859)	351 (222–555)	0.15	645 (324–1286)	240 (120–477)	*0*.*05*	485 (266–884)	556 (284–1088)	0.75
Pulsatile (ng/L/24 h)	609 (482–770)	786 (614–1007)	0.14	676 (490–933)	895 (649–1235)	0.21	556 (377–821)	657 (425–1016)	0.55
Total (ng/L/24 h)	1333 (1091–1629)	1235 (1000–1525)	0.60	1477 (1112–1965)	1234 (976–1639)	0.36	1206 (871–1669)	1234 (858–1774)	0.92
**Cortisol**									
Basal (nmol/L/24 h)	476 (216–1049)	708 (308–1631)	0.49	486 (146–1662)	721 (217–2392)	0.63	483 (133–1742)	662 (158–2774)	0.73
Pulsatile (nmol/L/24 h)	4487 (4000–5034)	4320 (3828–4880)	0.65	4298 (3678–5019)	4803 (4109–5608)	0.31	4708 (3971–5586)	3767 (3112–4555)	0.08
Total (nmol/L/24 h)	5481 (4803–6248)	5324 (4638–6118)	0.76	5351 (4452–6438)	5773 (4798–6940)	0.56	5631 (4583–6926)	4793 (3805–6039)	0.28

Data are presented as adjusted geometric mean with 95% confidence interval. Secretion rates were calculated with deconvolution analysis. Linear regression analyses were adjusted for age and BMI.

ACTH and cortisol parameters e.g. the slow half-life, pulse frequency, mean pulse mass and pulse mode during day and night were not different in offspring and controls, neither when men and women were combined nor when stratified for sex (S1 Fig).

There were no significant differences between offspring compared to controls in ACTH ApEn when men and women were combined (1.26 ± 0.07 *versus* 1.24 ± 0.07, *P* = 0.86) or in cortisol ApEn (1.07 ± 0.04 *versus* 1.13 ± 0.05, *P* = 0.34), nor when stratified for sex (Table 4).

**Table 4:** ApEn reflecting regularity of ACTH and cortisol secretory patterns and their cross-ApEn reflecting feedforward and feedback synchrony.

	All participants			Men			Women		
	Offspring n = 20	Controls n = 18	P-value	Offspring n = 10	Controlsn = 10	P-value	Offspring n = 10	Controlsn = 8	P-value
**ApEn**									
ACTH	1.26 (0.07)	1.24 (0.07)	0.86	1.29 (0.09)	1.11 (0.09)	0.22	1.23 (0.09)	1.41 (0.10)	0.18
Cortisol	1.07 (0.04)	1.13 (0.05)	0.34	1.07 (0.07)	1.12 (0.07)	0.62	1.08 (0.06)	1.16 (0.07)	0.36
**cross-ApEn**									
ACTH-Cortisol (feedforward)	1.41 (0.07)	1.39 (0.07)	0.84	1.40 (0.10)	1.23 (0.10)	0.24	1.42 (0.09)	1.60 (0.10)	0.19
Cortisol-ACTH (feedbackward)	1.33 (0.06)	1.29 (0.06)	0.67	1.34 (0.09)	1.21 (0.09)	0.31	1.31 (0.07)	1.40 (0.07)	0.37

Data are presented as mean with standard error of the mean (SEM). Linear regression analyses were adjusted for age and BMI.

### HPA-axis dynamics

ACTH-cortisol cross-ApEn, reflecting feedforward synchrony, was not different between offspring and partners when men and women were combined (1.41 ± 0.07 *versus* 1.39 ± 0.07, *P* = 0.84), nor when stratified for sex (Table 4). In addition, cortisol-ACTH cross-ApEn, reflecting feedback synchrony, was not significantly different in offspring and controls when men and women were combined (1.33 ± 0.06 *versus* 1.29 ± 0.06, *P* = 0.67), and also not when stratified for sex (Table 4).

**Fig 2:**
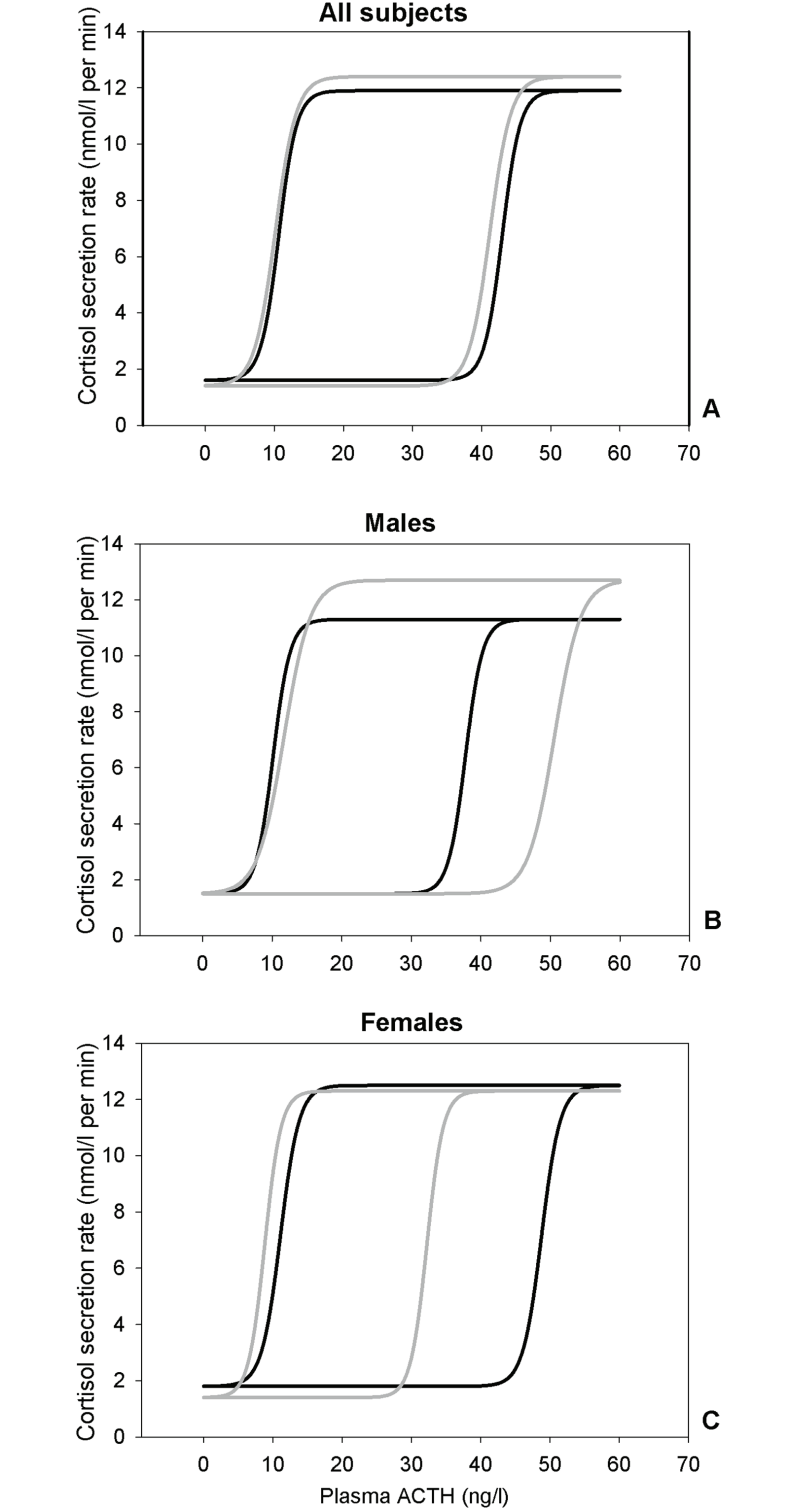
Adrenal gland responsivity to ACTH in all participants and stratified for sex. Adrenal gland responsivity to ACTH in an estimated endogenous ACTH-cortisol dose-response relationship in (A) 20 offspring (black line) and 18 controls (grey line). (B) 10 male offspring (black line) and 8 male controls (grey line). (C) 10 female offspring (black line) 10 female controls (grey line). In all panels, the left curves represent the dose-response during the initial phase of the secretory ACTH pulse, and the right curves represent the recovery phase, i.e. the decreasing part of the ACTH pulse, displaying the down-regulation.

The sensitivity of the adrenal gland for ACTH in both offspring and controls was assessed by modeling the endogenous ACTH-cortisol dose-response relationship (Fig 2). There were no differences in the endogenous ACTH-cortisol dose-response relationship between offspring and controls (Fig 2A). Male offspring compared to controls had a tendency towards a lower mean (95% CI) recovery EC_50_, but this did not reach statistical significance (38.0 (31.0–46.6) ng/L *versus* 50.5 (41.1–61.9) ng/L, *P* = 0.06) (Fig 2B). In women, there were no differences between groups in the endogenous ACTH-cortisol dose-response relationship (Fig 2C).

## Discussion

In this study, we investigated whether human longevity is associated with differences in HPA-axis regulation in offspring enriched for familial longevity compared to controls. We did not observe significant differences between offspring compared to controls in 24-h mean plasma concentrations of ACTH and serum concentrations of cortisol, or in their mean concentrations over 8 hr periods, although mean plasma ACTH concentrations tended to be non-significantly higher in female offspring compared to female controls over all time windows analyzed. In addition, male offspring had a higher basal ACTH secretion compared to male controls but no other differences were observed between groups in the deconvolution-derived 24-h secretion parameters of ACTH and cortisol. We did also not observe significant differences between groups in secretory regularity of ACTH and cortisol; in ACTH-cortisol feedforward and cortisol-ACTH feedback synchrony; or in endogenous ACTH-cortisol dose-response relationship, except for a trend towards a lower recovery EC_50_ in the endogenous ACTH-cortisol dose-response relationship in male offspring. These results suggest that familial longevity is not associated with major differences in the HPA-axis activity under resting conditions, although modest, sex-specific differences may exist between groups that might be clinically relevant.

Modest, sex-specific differences between groups included the trend towards a higher mean 24h ACTH in female offspring, and in males a significantly higher basal ACTH secretion in offspring and a borderline non-significant lower recovery EC_50_ in the endogenous ACTH-cortisol dose-response relationship. Although these measures reflect different features of the complex interplay between ACTH drive and cortisol output and feedback inhibition, these observations may hint at the existence of subtle, sex-specific differences between groups in the dynamics of the HPA axis. Previously, genetic polymorphisms in the glucocorticoid receptor which were associated in vivo with subtle differences in glucocorticoid feedback sensitivity have been associated with a more favorable glucose and lipid profiles as well as increased survival in a cohort of elderly men[26, 27]. Although a similar survival benefit was not found in another cohort of elderly[28], these results indicate that subtle changes in the dynamics of the HPA axis might have long-lasting clinical impact. Moreover, ACTH may have direct effects on metabolic tissues. Cold exposure activates the HPA axis, with ACTH having stimulatory and corticosterone having inhibitory effects on brown adipose tissue activity and browning of white adipose tissue[29]. Thus, subtle differences in the dynamic interplay between ACTH and cortisol may have pleiotropic effects on physiological processes beyond adrenal output that may have implications for metabolic health.

The HPA-axis can be modified by several factors, including age, sex, BMI, social economic status, chronic illness, psychiatric disorders and sleep disruption[30, 31]. Some studies have observed increased cortisol levels in aged compared to young participants[32–34] or in cortisol production rate[35], others found a potential age-dependent decline in total 24-h cortisol secretion[36] or no significant relationship between age and various measures of cortisol secretion[37–39]. No differences between young and aged participants were found in single ACTH levels during day and night[38] or in 24-h secretion rate[40], while one study found only in women age-related higher ACTH[41]. The two other important determinants of the HPA-axis are BMI and sex. Higher BMI leads to amplified ACTH and cortisol secretion, the latter without increased serum levels[35, 40, 42], while the influence of sex on the HPA-axis is less clear. One study found higher cortisol secretion in men[43], in other studies, women above 50 yr had higher cortisol levels than men[32]. Twenty-four hour secretion rate decreased by age in men, but increased in women[44], while no sex difference was found in two other studies[35, 45]. On the other hand, ACTH secretion is higher in men than women[40, 45]. In the present study, in which the two groups were comparable with respect to mean BMI, age and sex distribution, no differences in mean 24-h hormone levels were found, except for a slightly higher basal (but not total) ACTH secretion in male offspring. Thus, our hypothesis that longevity is associated with lower cortisol secretion was not confirmed in this study. The findings that there were no differences in cortisol secretion and mean cortisol levels were not in agreement with previous data of this cohort, where a slightly lower area under the curve (AUC) of morning and evening saliva cortisol was found in 149 offspring compared to 154 controls[14]. This contradictory result may be caused by differences in the analytical methods (saliva *versus* intensive blood sampling), in data analysis (AUC by four and two data points *versus* deconvolution-derived secretion rates based on 144 blood samples in each individual), in setting (home-based *versus* clinical setting), in the study sample size, or by differences in selection criteria on health of the participants[14]. Aging in the Brown Norway rat, who are long-living with a 50% survival beyond 2.5 year, is characterized by unchanged serum corticosterone levels with amplified ACTH secretion[46]. Long-lived Brown Norway rat[47] exhibit distinct differences in HPA-axis activity and reactivity. These include a faster recovery after restraint stress, larger adrenals that are less reactive to ACTH, higher efficiency of glucocorticoid receptor, and an apparent insensitivity to adrenalectomy. These later differences have been associated with genetic differences, amongst others in the mineralocorticoid receptor[48]. The Wistar Kyoto (WKY) rat is characterized by shorter life-span and hyper-reactivity to stressors[49]. Thus in rats, genetic differences in HPA-axis activity and reactivity have been associated with differences in lifespan.

The secretory regularity of ACTH in humans is age- and sex-independent[40], but obesity amplifies ACTH secretion, which is accompanied by decreased pattern regularity (increased ApEn)[50]. Cortisol secretion during normal aging becomes less regular, but not with obesity[40, 50]. Pattern synchrony, both feedforward and feedback, diminishes during aging[40]. In the present study, a comparable feedforward drive on cortisol, feedback on ACTH (respectively no differences in ApEn ACTH and Cortisol) and synchrony coupling between both hormone rhythms were found in offspring and controls (cross-ApEn ACTH cortisol and cross-ApEn cortisol ACTH). The finding of unchanged ACTH ApEn is also in line with previous research where we demonstrated no differences in cortisol feedback sensitivity between offspring enriched for longevity compared to controls, assessed by overnight dexamethasone suppression test and salivary cortisol levels the next morning in a home-based setting[14].

A new development in the HPA field is assessment of the endogenous ACTH-cortisol dose-response relationship, using prevailing physiological ACTH concentrations and resulting cortisol secretion rates[44]. Obesity in women is associated with decreased efficacy (maximal secretion) and sensitivity (slope), together with increased ED_50_, fully explaining non-increased serum cortisol levels in spite of increased plasma ACTH concentrations[24]. Increasing age and BMI diminishes sensitivity, while efficacy is increased in women, but decreased in men during aging. These changes in dose-response relationship tend to increase cortisol secretion in elderly women, in contrast to decreasing secretion in men, as found in some studies[32, 44]. In this study, no group and gender differences in efficacy or sensitivity of the adrenal gland to ACTH were observed.

In this study we were not able to detect major differences in HPA-axis in human enriched for familial longevity, although modest, sex-specific differences may exist between groups that might be clinically relevant. The moderate differences that we observed between groups have resulted in limited power to detect differences in parameters of the HPA axis between groups in the relatively small sample sizes that were available for the current study. The biggest difference observed between groups was a higher mean 24h ACTH concentration in female offspring compared to female controls. Given the observed mean (SD) 24h ACTH concentrations in female offspring and female controls, a sufficiently powered study (with significance of 0.05 and power of 80%) would have required double the sample size of the current study to detect significant differences between groups, namely 20 female offspring and 20 female controls. The observed differences between groups for mean (SD) 24h cortisol were even smaller and would thus have required even bigger sample sizes for sufficient power to detect significant differences between groups. Although the small study sample is a limitation of this study, the differences in age of the parents between offspring and controls is more than 12 years, which strongly suggests that our recruitment strategy to enrich for familial longevity was successful. Moreover, using the same sample size, we have been able to detect relatively large differences between groups in parameters of the hypothalamic-pituitary-thyroid (HPT)-axis, notably 60% higher mean 24-h concentrations of TSH[51]. Differences in the HPT-axis have been associated with longevity in animal models as well as in different human cohorts[52]. Future research should focus on disentangling differences between groups in acute rise in cortisol level in response to acute psychological stressors and physiological challenges.

## Supporting Information

S1 Table ACTH and cortisol secretion in all participants and stratified for sex. (DOCX)

S1 Fig Secretion parameters of the HPA-axis in all participants and stratified for sex. The black dots represents 20 offspring and the gray triangles represent 18 controls. Error bars represent standard error of the mean around the geometric mean. No significant differences between offspring and partners were presented.
(EPS)

S1 Dataset (XLSX)

## References

[1] Sapolsky RM, Krey LC, McEwen BS. The neuroendocrinology of stress and aging: the glucocorticoid cascade hypothesis. Endocrine reviews 1986;7(3):284–301. 10.1210/edrv-7-3-284 . 3527687

[2] McEwen BS. Protective and damaging effects of stress mediators. The New England journal of medicine 1998;338(3):171–9. 10.1056/NEJM199801153380307 . 9428819

[3] Bruehl H, Wolf OT, Convit A. A blunted cortisol awakening response and hippocampal atrophy in type 2 diabetes mellitus. Psychoneuroendocrinology 2009;34(6):815–21. 10.1016/j.psyneuen.2008.12.010 19167831 PMC2774914

[4] Wirtz PH, von Kanel R, Emini L, Ruedisueli K, Groessbauer S, Maercker A, et al Evidence for altered hypothalamus-pituitary-adrenal axis functioning in systemic hypertension: blunted cortisol response to awakening and lower negative feedback sensitivity. Psychoneuroendocrinology 2007;32(5):430–6. 10.1016/j.psyneuen.2007.02.006 . 17433557

[5] Walker BR, Phillips DI, Noon JP, Panarelli M, Andrew R, Edwards HV, et al Increased glucocorticoid activity in men with cardiovascular risk factors. Hypertension 1998;31(4):891–5. . 9535410 10.1161/01.hyp.31.4.891

[6] Walker BR, Soderberg S, Lindahl B, Olsson T. Independent effects of obesity and cortisol in predicting cardiovascular risk factors in men and women. Journal of internal medicine 2000;247(2):198–204. . 10692082 10.1046/j.1365-2796.2000.00609.x

[7] Phillips DI, Barker DJ, Fall CH, Seckl JR, Whorwood CB, Wood PJ, et al Elevated plasma cortisol concentrations: a link between low birth weight and the insulin resistance syndrome? The Journal of clinical endocrinology and metabolism 1998;83(3):757–60. 10.1210/jcem.83.3.4634 . 9506721

[8] Schoenmaker M, de Craen AJ, de Meijer PH, Beekman M, Blauw GJ, Slagboom PE, et al Evidence of genetic enrichment for exceptional survival using a family approach: the Leiden Longevity Study. European journal of human genetics: EJHG 2006;14(1):79–84. 10.1038/sj.ejhg.5201508 . 16251894

[9] Westendorp RG, van Heemst D, Rozing MP, Frolich M, Mooijaart SP, Blauw GJ, et al Nonagenarian siblings and their offspring display lower risk of mortality and morbidity than sporadic nonagenarians: The Leiden Longevity Study. Journal of the American Geriatrics Society 2009;57(9):1634–7. 10.1111/j.1532-5415.2009.02381.x . 19682117

[10] Stijntjes M, de Craen AJ, van Heemst D, Meskers CG, van Buchem MA, Westendorp RG, et al Familial longevity is marked by better cognitive performance at middle age: the Leiden Longevity Study. PloS one 2013;8(3):e57962 10.1371/journal.pone.0057962 23483953 PMC3587419

[11] Rozing MP, Westendorp RG, de Craen AJ, Frolich M, de Goeij MC, Heijmans BT, et al Favorable glucose tolerance and lower prevalence of metabolic syndrome in offspring without diabetes mellitus of nonagenarian siblings: the Leiden longevity study. Journal of the American Geriatrics Society 2010;58(3):564–9. 10.1111/j.1532-5415.2010.02725.x . 20398121

[12] Wijsman CA, Rozing MP, Streefland TC, le Cessie S, Mooijaart SP, Slagboom PE, et al Familial longevity is marked by enhanced insulin sensitivity. Aging cell 2011;10(1):114–21. 10.1111/j.1474-9726.2010.00650.x . 21070591

[13] Noordam R, Gunn DA, Tomlin CC, Rozing MP, Maier AB, Slagboom PE, et al Cortisol serum levels in familial longevity and perceived age: the Leiden longevity study. Psychoneuroendocrinology 2012;37(10):1669–75. 10.1016/j.psyneuen.2012.02.013 . 22429748

[14] Noordam R, Jansen SW, Akintola AA, Oei NY, Maier AB, Pijl H, et al Familial longevity is marked by lower diurnal salivary cortisol levels: the Leiden Longevity Study. PloS one 2012;7(2):e31166 10.1371/journal.pone.0031166 22348049 PMC3278433

[15] Fraser CG. Biological Variation: From Principles to Practice. Washington, DC: AACC Press; 2001.

[16] Liu PY, Keenan DM, Kok P, Padmanabhan V, O'Byrne KT, Veldhuis JD. Sensitivity and specificity of pulse detection using a new deconvolution method. American journal of physiology Endocrinology and metabolism 2009;297(2):E538–44. 10.1152/ajpendo.00071.2009 19531646 PMC2724108

[17] Keenan DM, Chattopadhyay S, Veldhuis JD. Composite model of time-varying appearance and disappearance of neurohormone pulse signals in blood. Journal of theoretical biology 2005;236(3):242–55. 10.1016/j.jtbi.2005.03.008 . 15916772

[18] Keenan DM, Roelfsema F, Biermasz N, Veldhuis JD. Physiological control of pituitary hormone secretory-burst mass, frequency, and waveform: a statistical formulation and analysis. American journal of physiology Regulatory, integrative and comparative physiology 2003;285(3):R664–73. 10.1152/ajpregu.00195.2003 . 12738612

[19] Veldhuis JD, Keenan DM, Pincus SM. Motivations and methods for analyzing pulsatile hormone secretion. Endocrine reviews 2008;29(7):823–64. 10.1210/er.2008-0005 18940916 PMC2647703

[20] Pincus SM. Irregularity and asynchrony in biologic network signals. Methods in enzymology 2000;321:149–82. . 10909056 10.1016/s0076-6879(00)21192-0

[21] Pincus S, Singer BH. Randomness and degrees of irregularity. Proceedings of the National Academy of Sciences of the United States of America 1996;93(5):2083–8. 11607637 10.1073/pnas.93.5.2083 PMC39913

[22] Veldhuis JD, Straume M, Iranmanesh A, Mulligan T, Jaffe C, Barkan A, et al Secretory process regularity monitors neuroendocrine feedback and feedforward signaling strength in humans. American journal of physiology Regulatory, integrative and comparative physiology 2001;280(3):R721–9. . 11171650 10.1152/ajpregu.2001.280.3.R721

[23] Keenan DM, Licinio J, Veldhuis JD. A feedback-controlled ensemble model of the stress-responsive hypothalamo-pituitary-adrenal axis. Proceedings of the National Academy of Sciences of the United States of America 2001;98(7):4028–33. 10.1073/pnas.051624198 11274427 PMC31173

[24] Roelfsema F, Pijl H, Keenan DM, Veldhuis JD. Diminished adrenal sensitivity and ACTH efficacy in obese premenopausal women. European journal of endocrinology / European Federation of Endocrine Societies 2012;167(5):633–42. 10.1530/EJE-12-0592 . 22909443

[25] Keenan DM, Roelfsema F, Veldhuis JD. Dose-response downregulation within the span of single interpulse intervals. American journal of physiology Regulatory, integrative and comparative physiology 2010;299(1):R11–8. 10.1152/ajpregu.00201.2010 20410472 PMC2904156

[26] van Rossum EF, Feelders RA, van den Beld AW, Uitterlinden AG, Janssen JA, Ester W, et al Association of the ER22/23EK polymorphism in the glucocorticoid receptor gene with survival and C-reactive protein levels in elderly men. The American journal of medicine 2004;117(3):158–62. 10.1016/j.amjmed.2004.01.027 . 15276593

[27] van Rossum EF, Koper JW, Huizenga NA, Uitterlinden AG, Janssen JA, Brinkmann AO, et al A polymorphism in the glucocorticoid receptor gene, which decreases sensitivity to glucocorticoids in vivo, is associated with low insulin and cholesterol levels. Diabetes 2002;51(10):3128–34. . 12351458 10.2337/diabetes.51.10.3128

[28] Kuningas M, Mooijaart SP, Slagboom PE, Westendorp RG, van Heemst D. Genetic variants in the glucocorticoid receptor gene (NR3C1) and cardiovascular disease risk. The Leiden 85-plus Study. Biogerontology. 2006;7(4):231–8. 10.1007/s10522-006-9021-2 . 16676134

[29] van den Beukel JC, Grefhorst A, Quarta C, Steenbergen J, Mastroberardino PG, Lombes M, et al Direct activating effects of adrenocorticotropic hormone (ACTH) on brown adipose tissue are attenuated by corticosterone. FASEB journal: official publication of the Federation of American Societies for Experimental Biology 2014;28(11):4857–67. 10.1096/fj.14-254839 . 25085924

[30] Seeman T, Epel E, Gruenewald T, Karlamangla A, McEwen BS. Socio-economic differentials in peripheral biology: cumulative allostatic load. Annals of the New York Academy of Sciences 2010;1186:223–39. 10.1111/j.1749-6632.2009.05341.x . 20201875

[31] Veldhuis JD, Sharma A, Roelfsema F. Age-dependent and gender-dependent regulation of hypothalamic-adrenocorticotropic-adrenal axis. Endocrinology and metabolism clinics of North America 2013;42(2):201–25. 10.1016/j.ecl.2013.02.002 23702398 PMC3675779

[32] Van Cauter E, Leproult R, Kupfer DJ. Effects of gender and age on the levels and circadian rhythmicity of plasma cortisol. The Journal of clinical endocrinology and metabolism 1996;81(7):2468–73. 10.1210/jcem.81.7.8675562 . 8675562

[33] Halbreich U, Asnis GM, Zumoff B, Nathan RS, Shindledecker R. Effect of age and sex on cortisol secretion in depressives and normals. Psychiatry research 1984;13(3):221–9. . 6597461 10.1016/0165-1781(84)90037-4

[34] Deuschle M, Gotthardt U, Schweiger U, Weber B, Korner A, Schmider J, et al With aging in humans the activity of the hypothalamus-pituitary-adrenal system increases and its diurnal amplitude flattens. Life sciences 1997;61(22):2239–46. . 9393943 10.1016/s0024-3205(97)00926-0

[35] Purnell JQ, Brandon DD, Isabelle LM, Loriaux DL, Samuels MH. Association of 24-hour cortisol production rates, cortisol-binding globulin, and plasma-free cortisol levels with body composition, leptin levels, and aging in adult men and women. The Journal of clinical endocrinology and metabolism 2004;89(1):281–7. 10.1210/jc.2003-030440 . 14715862

[36] Sharma M, Palacios-Bois J, Schwartz G, Iskandar H, Thakur M, Quirion R, et al Circadian rhythms of melatonin and cortisol in aging. Biological psychiatry 1989;25(3):305–19. . 2914154 10.1016/0006-3223(89)90178-9

[37] Sherman B, Wysham C, Pfohl B. Age-related changes in the circadian rhythm of plasma cortisol in man. The Journal of clinical endocrinology and metabolism 1985;61(3):439–43. 10.1210/jcem-61-3-439 . 4019712

[38] Waltman C, Blackman MR, Chrousos GP, Riemann C, Harman SM. Spontaneous and glucocorticoid-inhibited adrenocorticotropic hormone and cortisol secretion are similar in healthy young and old men. The Journal of clinical endocrinology and metabolism 1991;73(3):495–502. 10.1210/jcem-73-3-495 . 1651956

[39] Touitou Y, Sulon J, Bogdan A, Reinberg A, Sodoyez JC, Demey-Ponsart E. Adrenocortical hormones, ageing and mental condition: seasonal and circadian rhythms of plasma 18-hydroxy-11-deoxycorticosterone, total and free cortisol and urinary corticosteroids. The Journal of endocrinology 1983;96(1):53–64. . 6822782 10.1677/joe.0.0960053

[40] Veldhuis JD, Roelfsema F, Iranmanesh A, Carroll BJ, Keenan DM, Pincus SM. Basal, pulsatile, entropic (patterned), and spiky (staccato-like) properties of ACTH secretion: impact of age, gender, and body mass index. The Journal of clinical endocrinology and metabolism 2009;94(10):4045–52. 10.1210/jc.2009-1143 19755477 PMC2758736

[41] Ferrari E, Magri F, Dori D, Migliorati G, Nescis T, Molla G, et al Neuroendocrine correlates of the aging brain in humans. Neuroendocrinology 1995;61(4):464–70. . 7783860 10.1159/000126869

[42] Roelfsema F, Kok P, Frolich M, Pereira AM, Pijl H. Disordered and increased adrenocorticotropin secretion with diminished adrenocorticotropin potency in obese in premenopausal women. The Journal of clinical endocrinology and metabolism 2009;94(8):2991–7. 10.1210/jc.2009-0350 . 19454578

[43] Vierhapper H, Nowotny P, Waldhausl W. Production rates of cortisol in men with hypogonadism. Metabolism: clinical and experimental 2004;53(9):1174–6. . 15334380 10.1016/j.metabol.2004.02.021

[44] Keenan DM, Roelfsema F, Carroll BJ, Iranmanesh A, Veldhuis JD. Sex defines the age dependence of endogenous ACTH-cortisol dose responsiveness. American journal of physiology Regulatory, integrative and comparative physiology 2009;297(2):R515–23. 10.1152/ajpregu.00200.2009 19535673 PMC2724232

[45] Veldhuis JD, Iranmanesh A, Roelfsema F, Aoun P, Takahashi P, Miles JM, et al Tripartite control of dynamic ACTH-cortisol dose responsiveness by age, body mass index, and gender in 111 healthy adults. The Journal of clinical endocrinology and metabolism 2011;96(9):2874–81. 10.1210/jc.2011-0084 21752885 PMC3167672

[46] van Eekelen JA, Rots NY, Sutanto W, de Kloet ER. The effect of aging on stress responsiveness and central corticosteroid receptors in the brown Norway rat. Neurobiol Aging 1992;13(1):159–70. . 1311803 10.1016/0197-4580(92)90024-r

[47] Mos J, Hollander CF. Analysis of survival data on aging rat cohorts: pitfalls and some practical considerations. Mechanisms of ageing and development 1987;38(1):89–105. . 3600047 10.1016/0047-6374(87)90113-8

[48] Marissal-Arvy N, Lombes M, Petterson J, Moisan MP, Mormede P. Gain of function mutation in the mineralocorticoid receptor of the Brown Norway rat. The Journal of biological chemistry 2004;279(38):39232–9. 10.1074/jbc.M407436200 . 15252022

[49] Tizabi Y, Aguilera G, Gilad GM. Age-related reduction in pituitary corticotropin-releasing hormone receptors in two rat strains. Neurobiology of aging 1992;13(2):227–30. . 1326090 10.1016/0197-4580(92)90034-u

[50] Kok P, Roelfsema F, Frolich M, van Pelt J, Meinders AE, Pijl H. Bromocriptine reduces augmented thyrotropin secretion in obese premenopausal women. The Journal of clinical endocrinology and metabolism 2009;94(4):1176–81. 10.1210/jc.2008-2303 . 19190107

[51] Jansen SW, Akintola AA, Roelfsema F, van der Spoel E, Cobbaert CM, Ballieux BE, et al Human longevity is characterised by high thyroid stimulating hormone secretion without altered energy metabolism. Sci Rep 2015;5 10.1038/srep11525 PMC4473605 26089239

[52] Bowers J, Terrien J, Clerget-Froidevaux MS, Gothie JD, Rozing MP, Westendorp RG, et al Thyroid hormone signaling and homeostasis during aging. Endocrine reviews 2013;34(4):556–89. 10.1210/er.2012-1056 . 23696256

