# Supplementary material for: Characterization of the Hypothalamic-Pituitary-Adrenal-Axis in Familial Longevity under Resting Conditions
Source: PLoS One. 2015 Jul 20;10(7):e0133119. doi: 10.1371/journal.pone.0133119 (PMC4508039; doi:10.1371/journal.pone.0133119)
Supplement: S1 Table — (DOCX) [file pone.0133119.s001.docx]

**S1 Table.** **ACTH and cortisol secretion in all participants and stratified for sex.**

|  | **All participants** | |  | **Men** | |  | **Women** | |  |
| --- | --- | --- | --- | --- | --- | --- | --- | --- | --- |
|  | Offspring n=20 | Controls n=18 | P-value | Offspring n=10 | Controls n=10 | P -value | Offspring n=10 | Controls n=8 | P -value |
| **ACTH** |  |  |  |  |  |  |  |  |  |
| *Basal (ng/L)* |  |  |  |  |  |  |  |  |  |
| 0900-1700 h | 246 (178-340) | 235 (167-330) | 0.84 | 258 (164-405) | 233 (149-367) | 0.74 | 234 (139-396) | 236 (131-425) | 0.99 |
| 1700-0000 h | 193 (99-269) | 85 (50-144) | 0.08 | 145 (58-365) | 72 (29-182) | 0.28 | 183 (113-297) | 104 (61-179) | 0.12 |
| 0000-0800 h | 191 (118-310) | 114 (69-190) | 0.15 | 250 (110-565) | 98 (44-223) | 0.11 | 147 (83-261) | 138 (72-2847) | 0.88 |
| *Pulsatile (ng/L)* |  |  |  |  |  |  |  |  |  |
| 0900-1700 h | 185 (143-241) | 167 (127-219) | 0.75 | 183 (124-269) | 237 (161-349) | 0.33 | 188 (141-251) | 107 (78-148) | 0.02 |
| 1700-0000 h | 163 (128-209) | 131 (101-170) | 0.23 | 142 (102-197) | 150 (108-209) | 0.80 | 163 (128-209) | 131 (101-170) | 0.23 |
| 0000-0800 h | 265 (199-352) | 279 (207-377) | 0.80 | 284 (186-435) | 401 (262-613) | 0.25 | 246 (178-341) | 178 (124-255) | 0.17 |
| *Total (ng/L)* |  |  |  |  |  |  |  |  |  |
| 0900-1700 h | 461 (368-577) | 443 (356-561) | 0.80 | 471 (339-653) | 500 (360-694) | 0.79 | 452 (322-633) | 380 (261-554) | 0.48 |
| 1700-0000 h | 364 (283-447) | 250 (192-325) | 0.04 | 337 (229-497) | 266 (180-392) | 0.37 | 392 (274-561) | 231 (155-344) | 0.053 |
| 0000-0800 h | 483 (376-620) | 495 (380-644) | 0.89 | 558 (394-789) | 658 (465-931) | 0.49 | 417 (302-577) | 346 (241-497) | 0.43 |
| **Cortisol** |  |  |  |  |  |  |  |  |  |
| *Basal (nmol/L)* |  |  |  |  |  |  |  |  |  |
| 0900-1700 h | 468 (196-1118) | 186 (75-466) | 0.15 | 662 (157-2793) | 88 (21-370) | 0.052 | 332 (127-863) | 479 (164-1395) | 0.60 |
| 1700-0000 h | 257 (86-714) | 95 (31-292) | 0.22 | 109 (38-308) | 476 (168-1349) | 0.05 | 563 (125-2540) | 13 (2-69) | 0.03 |
| 0000-0800 h | 81 (22-307) | 70 (17-283) | 0.87 | 69 (8-580) | 38 (5-319) | 0.68 | 95 (16-580) | 148 (20-931) | 0.73 |
| *Pulsatile (nmol/L)* |  |  |  |  |  |  |  |  |  |
| 0900-1700 h | 1171 (932-1473) | 1140 (895-1451) | 0.87 | 1036 (757-1419) | 1258 (920-1723) | 0.37 | 1325 (923-1899) | 1006 (673-1506) | 0.30 |
| 1700-0000 h | 781 (572-1066) | 800 (577-1111) | 0.91 | 687 (432-1092) | 643 (405-1021) | 0.83 | 887 (572-1375) | 1053 (645-1716) | 0.59 |
| 0000-0800 h | 2115 (1808-2475) | 2222 (1882-2623) | 0.67 | 2310 (2055-2597) | 2423 (2156-2724) | 0.55 | 1939 (1420-2643) | 1992 (1408-2818) | 0.90 |
| *Total (nmol/L)* |  |  |  |  |  |  |  |  |  |
| 0900-1700 h | 1858 (1544-2237) | 1720 (1415-2092) | 0.57 | 1767 (1411-2213) | 1848 (1476-2315) | 0.77 | 1955 (1411-2708) | 1572 (1092-2264) | 0.36 |
| 1700-0000 h | 1246 (948-1637) | 1190 (892-1588) | 0.82 | 977 (677-1407) | 1138 (789-1641) | 0.54 | 1589 (1042-2426) | 1260 (785-2124) | 0.45 |
| 0000-0800 h | 2799 (2443-3207) | 2468 (2139-2847) | 0.21 | 2744 (2371-3172) | 2711 (2345-3137) | 0.91 | 2855 (2231-3652) | 2193 (1666-2890) | 0.15 |

Data are presented as geometric mean with 95% confidence interval.
Statistical significant was calculated with linear regression analyses.
Secretion rates were calculated with deconvolution analysis
